# Relationship between lateral differences in the cross-sectional area of the psoas muscle and curve running time

**DOI:** 10.1186/s40101-016-0086-6

**Published:** 2016-01-26

**Authors:** Nobuaki Tottori, Toshiyuki Kurihara, Mitsuo Otsuka, Tadao Isaka

**Affiliations:** Department of Sport and Health Science, Ritsumeikan University, Kusatsu, Shiga Japan

**Keywords:** Track and field athlete, Counterclockwise direction, Magnetic resonance imaging, Hip muscle, Thigh muscle

## Abstract

**Background:**

The aim of this study was to investigate whether lateral differences in the cross-sectional areas of the hip and thigh muscles were related to curve sprinting time.

**Methods:**

Thirteen college students (10 men and 3 women; mean ± SD: age, 20.4 ± 1.7 years; height, 167.6 ± 8.9 cm; mass, 57.4 ± 5.4 kg) participated in this study. The participants were instructed to sprint along a circular track (23 m radius) in the counterclockwise and clockwise directions. Magnetic resonance imaging was used to measure the cross-sectional area of the psoas major, quadriceps femoris, and hamstring muscles. The symmetry index was used to evaluate the lateral differences in the cross-sectional area.

**Results:**

The lateral difference was observed in the cross-sectional area (CSA) of the thigh muscles, not in the psoas major muscle. The sprint time was not significantly different between the counterclockwise (22.15 ± 2.27 s) and clockwise (22.13 ± 2.32 s) directions. No significant correlations were found between the symmetry index of the thigh muscles and the cross-directional differences in sprint time. However, the symmetry index of the psoas major muscle correlated significantly with the cross-directional difference in sprint time (*r* = −0.614, *P* = 0.026).

**Conclusions:**

These findings suggest that the participants in whom the cross-sectional area of the psoas major muscle of the outer leg was larger than that of the inner leg were faster in curve sprinting.

## Introduction

Many studies have investigated running, but few studies have focused on running along a curved path [1–6]. Besides the 100-m race, all track competitions of the International Association of Athletics Federations [7] include a period of curve running. A study on sprint running along a curved path is important because in the 400-m standard track, the curved distance travelled is longer than the distance travelled on a straight path. It is generally accepted that sprint running performance on a curved path is inferior to that on a straight path. Ryan et al. [8] reported that the difference in the time taken to complete a 100-m run along a straight path and a curved path ranged from 0.177 to 0.400 s [1–3, 9–11].

Leaning the whole body into the curve is believed to cause the detrimental effect observed during curve running, as the sprinter must generate additional centripetal force in the lateral direction [12]. Some previous studies demonstrated the lateral differences in peak vertical ground reaction force and hip extension velocity during curve running [4, 5]. The peak vertical ground reaction force of the outer leg is larger than that of the inner leg [4], and the maximal hip extension velocity of the inner leg is significantly lower in curved running than in straight running [5]. In addition, the braking impulse of the inner leg is larger than that of the outer leg due to longer ground contact time of the inner leg, and the peak inward ground reaction force of the inner leg is larger than that of the outer leg [2]. As for the walking along a circular path, the pronation moment of the inner leg and the supination moment of the outer leg increase with the walking velocity [13], which requires an increase in the stabilization of the non-sagittal plane. These results suggest that running performance along a curve is influenced by the muscle force differences between the inner and outer legs.

Track and field athletes run counterclockwise repetitively on a curve during daily training. According to IAAF regulations [7], the direction of running around a track is designated as counterclockwise. Because of IAAF regulations [7], this training may cause the functional and/or morphological characteristics of the muscles to adapt to the specific purposes of the athletes’ events. Previous studies showed a significant correlation between the psoas major (PM) muscle size and sprint performance [14]. Alternatively, Hoshikawa et al. [15] found a positive correlation between the mean sprinting velocity and the PM to quadriceps femoris (QF) ratio obtained considering their cross-sectional area (CSA). In addition, considering that sprinters are known to have significantly greater QF and hamstrings (HM) CSA than non-athletes [16, 17], the CSA of the QF and HM should also be important for sprinting performance.

The purpose of this study was to investigate whether lateral differences in the CSA between the hip and thigh muscles was related to the curve sprinting time of track and field athletes. We hypothesized that runners adapt their hip and thigh muscles to specific purposes; therefore, lateral differences would be observed in the CSA of the muscles. This lateral difference would allow for faster sprint running in the counterclockwise direction than in the clockwise direction.

## Methods

### Participants

Thirteen healthy college students volunteered to participate in the study (10 men and 3 women; mean ± standard deviation: age, 20.4 ± 1.7 years; body height, 167.6 ± 8.9 cm; body mass, 57.4 ± 5.4 kg). All participants had undergone at least 4 years of track and field training and were engaged in regular training in the counterclockwise direction for 4 days per week. The participants had no hip or lower limb injuries during the 6 months prior to the experiment. Ten participants specialized in middle-long distance running events (average 5000 m best time, 15 min 50 s; average 1500 m best time, 4 min 21 s), and three specialized in sprint events (average 400 m best time, 52.1 s). All participants were right leg dominant as determined by procedures defined in a previous report [18]. In short, the dominant leg was evaluated by their foot preference in 11 items of inquiry. In accordance with the Declaration of Helsinki, the experimental procedure was explained to each participant who then signed a written informed consent. The study was approved by the local ethics committee of Ritsumekan University.

### Experimental procedure

**Fig. 1:**
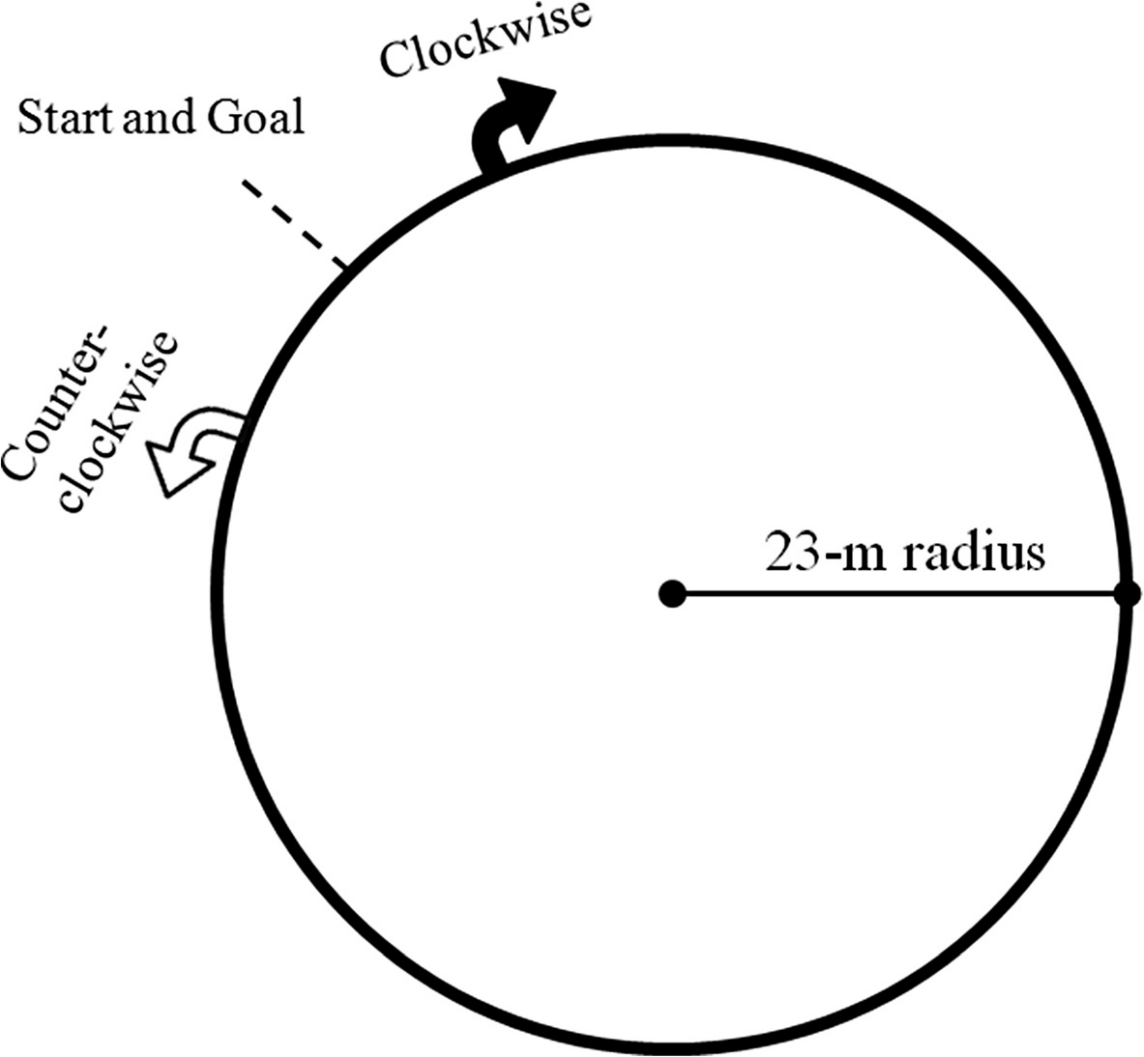
Overhead view of the experimental set-up. The geometry of a 23-m radius circle was drawn on flat dirt ground

The participants were instructed to sprint one trial of each counterclockwise and clockwise direction with maximum effort along a circular track after sufficient warm-up. These trial orders were randomized. To maximize their performance on the circular track in both directions without wearing spike shoes, the participants were allowed to run with sub-maximum effort several times. A 23-m-radius circular track, with a curvature representing lane six of the 200-m standard indoor track (circumference, 144 m) of the IAAF, was drawn on flat clay ground (Fig. 1). The sprint time for one round of the circular track was recorded by one person who has a credential for track and field referee with a stopwatch in 1/100 s. After each trial, the participants were asked to rate their perceived performance using a 5-point scale (1 = worst performance, 5 = best performance). A 15-min rest period was allowed between trials to minimize fatigue.

**Fig. 2:**
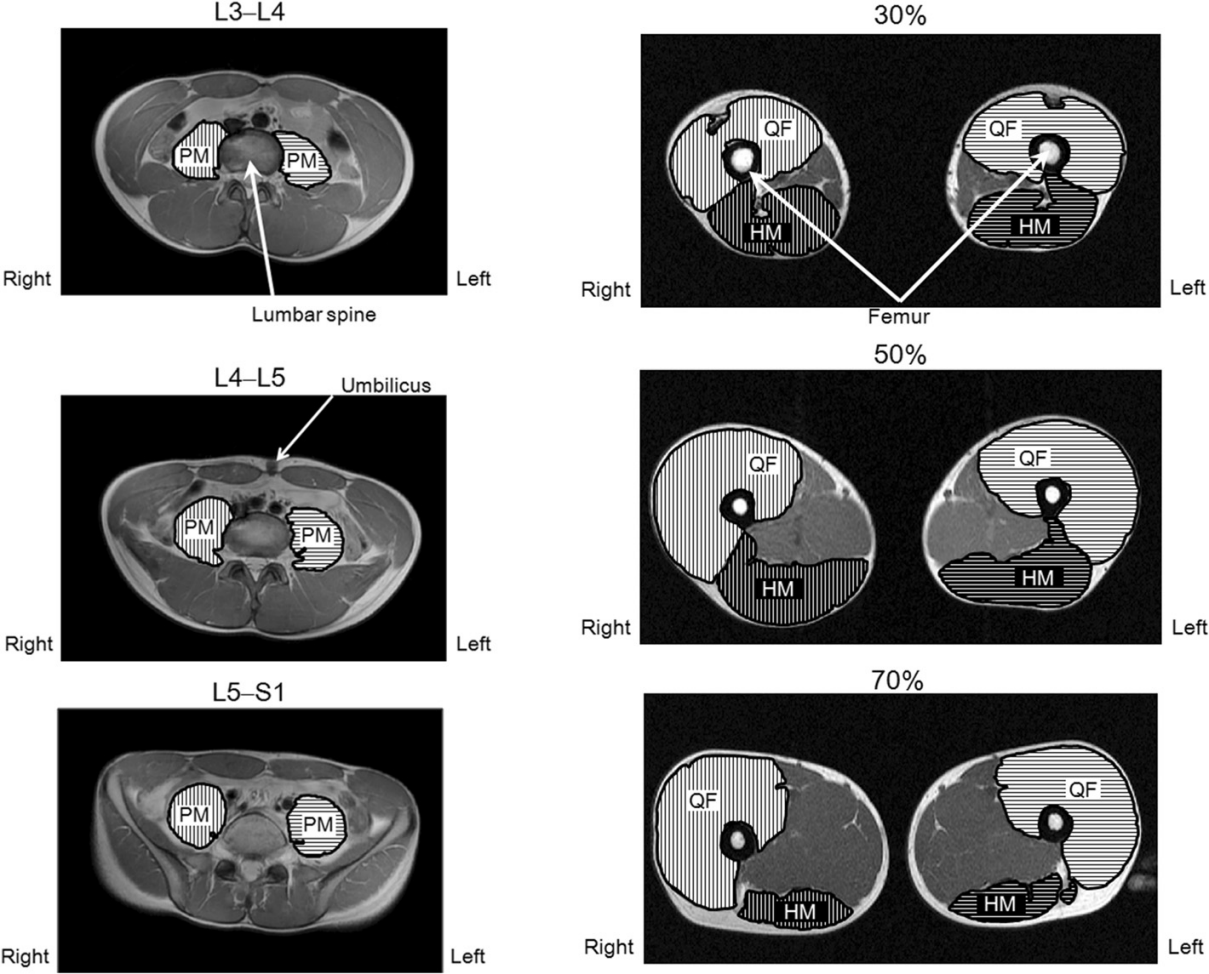
Typical cross-sectional magnetic resonance images of the psoas major muscle outlined at the L3–L4, L4–L5, and L5–S1 levels (L: lumbar spine, S: sacral spine), and those of the thigh muscles at the proximal 30, 50, and 70 % of the femur length

The CSA of the PM, QF, and HM muscles (including the semitendinosus, semimembranosus, and biceps femoris muscles) on both sides were measured with magnetic resonance imaging (MRI). A 1.5-T whole body scanner (SignaHDxt, GE Healthcare UK Ltd, Buckinghamshire, England) was used to acquire the MR images. The participants were scanned in the supine position with the hip and knee joints extended. Transverse T1-weighted MR images were obtained (repetition time, 600 ms; echo time, 7.7 ms; slice thickness, 10 mm; interspaced distance, 0 mm; field of view, 420 × 420 mm; matrix, 512 × 512). The images at the midlevel of L3–L4, L4–L5, and L5–S1 (L: lumbar spine, S: sacral spine) and at the proximal 30, 50, and 70 % of the femur length (0 %, lateral condyle of the femur; 100 %, greater trochanter) were selected to determine the CSA of the PM, QF, and HM muscles (Fig. 2). All measurements and calculations were performed by the same investigator (N.T.) with specifically designed image analysis software (SliceOmatic 4.3, Tomovision Inc., Montreal, Canada). In short, a threshold was selected on the basis of the grey-level image pixel, and the tissue boundaries were manually traced. The PM, QF, and HM muscles were measured separately. Intra-rater reliability of manual tracing was assessed with the use of intraclass correlation coefficients (ICCs), and elucidated excellent (ICC = 0.99).

### Data analysis

The cross-directional differences in sprint time were calculated by subtracting the clockwise direction sprint time from the counterclockwise direction sprint time:

Cross-directional difference in sprint time (%) = {[sprint time for the counterclockwise direction (s)] − [sprint time for the clockwise direction (s)]} / [sprint time for the clockwise direction (s)] * 100.

A positive value of the cross-directional difference in sprint time indicated that the sprint for the counterclockwise direction was slower than that for the clockwise direction.

The lateral difference in muscle size at each level, and the sum were evaluated and considered the symmetry index [19] using the following equation:

$$ \mathrm{Symmetry}\ \mathrm{index}\ \left(\%\right) = \left[2\ *\ \left(\mathrm{right}\ \mathrm{side} - \mathrm{left}\ \mathrm{side}\right)/\left(\mathrm{right}\ \mathrm{side} + \mathrm{left}\ \mathrm{side}\right)\right]\ *\ 100. $$

A positive value of the symmetry index indicated that the right side of the muscle was larger than the left side of the muscle.

The sum of the CSA of the PM, QF, and HM at three levels was calculated as the index of muscle volume. Total muscle volumes were calculated to put together left and right sum of CSA.

### Statistical analysis

Dependent variables included the CSA at each level and the sum of the PM, QF, and HAM. A paired sample *t* test was used to determine if the differences existed between the sides. The Pearson correlation coefficient was used to assess the relationship between the cross-directional differences in sprint time and symmetry indices, between averaged sprint time along a curve and total muscle volume, symmetry indices. The level of significance was set at *P* < 0.05, and descriptive data were presented as mean ± SD.

## Results

### Laterality in sprint time and muscular balance

No significant differences in sprint time were observed between the counterclockwise (22.15 ± 2.27 s) and clockwise directions (22.13 ± 2.32 s). According to the self-rated performance, the participants felt their sprinting performance better in the counterclockwise direction than in the clockwise direction (counterclockwise = 4.07 ± 1.07 points, clockwise = 2.36 ± 0.63 points, *P* < 0.01).

**Fig. 3:**
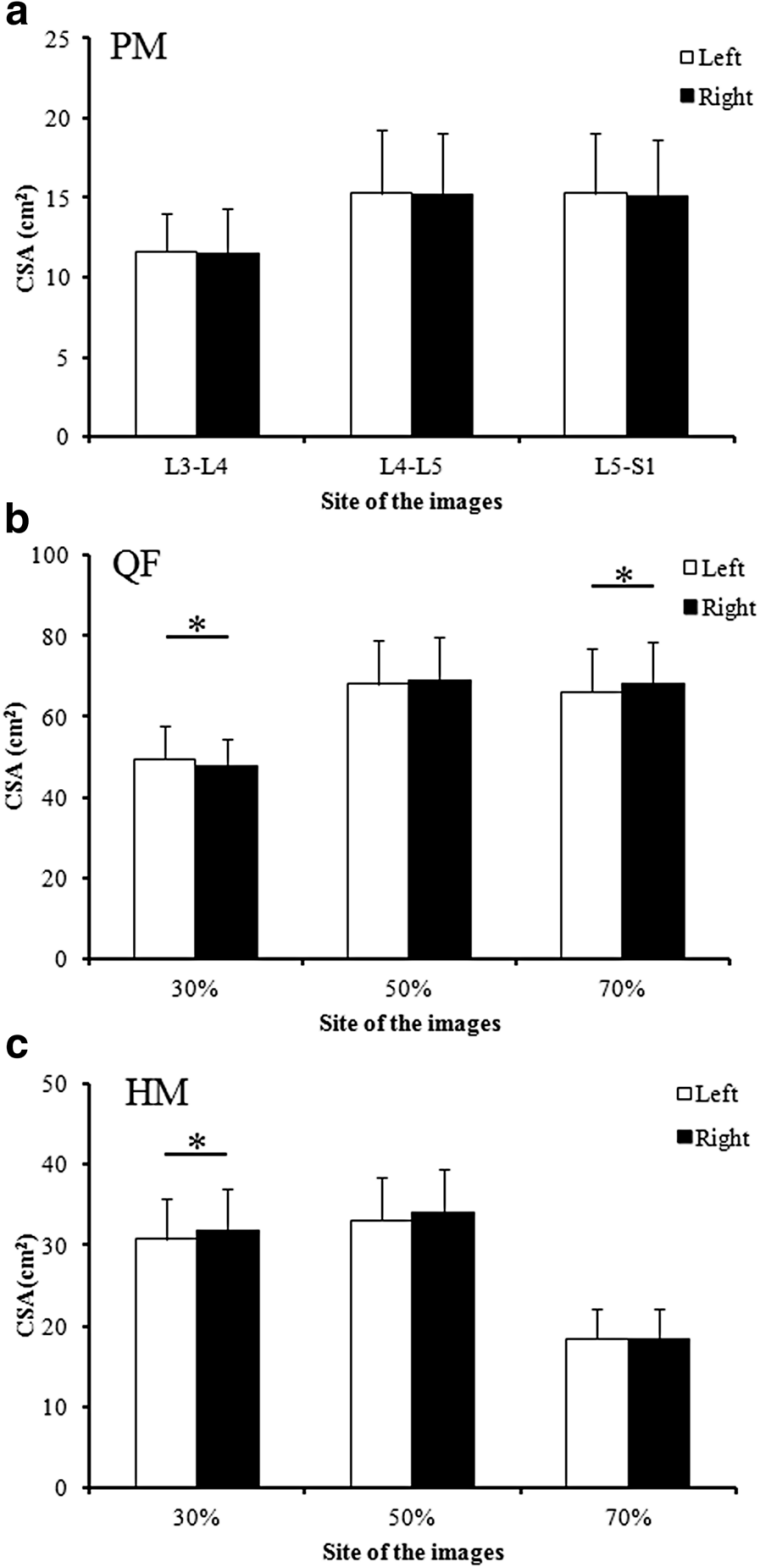
CSA of the muscles psoas major (**a**), quadriceps femoris (**b**), and hamstrings (**c**) on both sides of each measurement site. *significant difference between sides at *P* < 0.05. *L* lumbar spine, *S* sacral spine, *30 %*, *50 %*, and *70 %* proximal 30, 50, and 70 % of the femur length, *PM* psoas major muscle, *QF* quadriceps femoris muscles, *HM* = hamstring muscles

No significant lateral differences in the CSA of the PM muscle were found at any level (Fig. 3a). Meanwhile, the CSA of the left QF muscle at 30 % of the femoral length was significantly greater than that of the right QF muscle, and the CSA of the right QF muscle at 70 % of the femoral length was significantly greater than that of the left QF muscle (Fig. 3b). Moreover, the CSA of the right HM muscle at 30 % of the femoral length was significantly greater than that of the left HM muscle (Fig. 3c).

### Relationship between the lateral difference in the muscle and cross-directional difference in sprint time

**Fig. 4:**
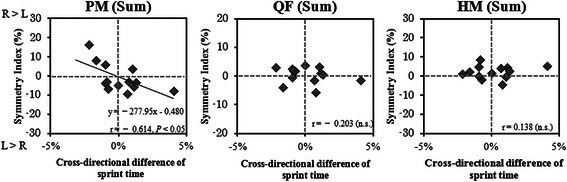
Relationships between cross-directional difference in sprint time and symmetry index of the sum of psoas major (PM), quadriceps femoris (QF), and hamstrings (HM)

The symmetry index of the sum of the PM muscle significantly correlated with the cross-directional difference in sprint time (*r* = −0.614, *P* < 0.05; Table 1, Fig. 4). No significant correlation was observed between the symmetry index of the L3–L4, L4–L5, or L5–S1 levels and cross-directional differences in sprint time (Table 1). The symmetry index of the QF and HM muscles in the entire femur length was not significantly correlated with the cross-directional difference in sprint time (Table 1, Fig. 4).

**Table 1:** Coefficients of correlation, coefficients of determination, and probability of the relationship between cross-directional difference in sprint time and symmetry index of each CSA

		*r*	*R* ^2^	*P*
PM	L3–L4	−0.541	0.29	0.056
	L4–L5	−0.550	0.30	0.052
	L5–S1	−0.495	0.25	0.084
	SUM	−0.614	0.38	0.026*
QF	30 %	−0.062	0.00	0.839
	50 %	−0.399	0.16	0.177
	70 %	−0.054	0.00	0.860
	SUM	−0.203	0.04	0.506
HM	30 %	0.292	0.09	0.333
	50 %	0.190	0.04	0.535
	70 %	−0.275	0.08	0.363
	SUM	0.138	0.02	0.654

*Indicate that correlation is significant at *P* < 0.05

*PM* psoas major, *QF* quadriceps femoris, *HM* hamstrings

### Relationship between averaged sprint time along a curve and muscle volume

Averaged sprint time along a curve with counterclockwise and clockwise directions significantly correlated with total muscle volume of PM, QF, and HM (Table 2). On the other hand, no significant correlation was observed between averaged sprint time along a curve and symmetry index of sum of these muscles (Table 2).

**Table 2:** Coefficients of correlation, coefficients of determination and probability of the relationship between averaged sprint time and total muscle volumes and symmetry index of each muscle

		*r*	*R* ^2^	*P*
PM	Volume	−0.859	0.74	<0.001***
	Symmetry	0.092	0.01	0.765
QF	Volume	−0.792	0.63	<0.001***
	Symmetry	0.415	0.17	0.158
HM	Volume	0.827	0.68	<0.001***
	Symmetry	0.064	0.00	0.836

***Indicate that correlation is significant at *P* < 0.001

*PM* psoas major, *QF* quadriceps femoris, *HM* hamstrings

## Discussion

The purpose of this study was to investigate the relationship between the lateral differences in the CSA of the hip and thigh muscles and the curve sprinting time of track and field athletes. The lateral difference was observed in the CSA of QF and HM as with the initial hypothesis. However, no significant difference was found in the CSA of the PM. No significant differences were observed in sprint running time between the counterclockwise and clockwise directions. Contrary to our hypothesis, no significant correlation was observed between the lateral differences in the CSA of the QF and HM muscles and the cross-directional difference in curve sprinting time. Whereas, the lateral difference in the PM size significantly correlated with the cross-directional difference in curve sprinting time. In short, the participants in the present study with a larger PM on the right side ran faster in the counterclockwise direction than in the clockwise direction. In other words, individuals with a larger CSA of the PM in the outer leg could be effective in curve running. The *R* ^2^ values were 0.38, so this is expected since the iliopsoas muscle on the outside is important for curve running.

The lateral difference of the CSA of QF and HM were observed of track and field athletes. These results support the initial hypothesis that track and field athletes have adapted the muscles for specific purpose of sprinting along a curve. The outer leg exerts a larger magnitude of force onto the ground than the inner leg in curve running [4]. Moreover, anteroposterior propulsive impulse during for the outer leg during curve running was higher than straight but not for the inner leg [3]. However, the present study demonstrated that the symmetry indices of the QF and HM muscles were not significantly correlated with the cross-directional difference in sprint time (Table 1). From a model-based calculation, the knee flexion torque on the swing leg significantly increased during the terminal swing phase [20, 21]. However, the kinematic analysis of a previous study, Alt et al. [6], indicated that the knee joint angles were not different between the inner and outer legs, while the hip and ankle joint angles were different between the legs. In addition, from the model calculation of a previous study [20], the knee flexion torque during the latter half of the swing phase was mainly produced by the ipsilateral HM muscles, but at the same time, the HM muscles in the supporting leg contributed to contralateral knee extension torque during the first half of the swing phase. These diverse effects could balance out the effects of a larger CSA of the outer and inner HM muscles on sprint performance during curve running. Therefore, the lateral difference in the QF and HM muscles may not affect the curve running time.

Significant lateral differences were not observed in the CSAs of the PM muscle of track and field athletes. This result is in contrast to our initial hypothesis that track and field athletes have adapted their muscles for the specific purpose of sprinting faster in the counterclockwise direction. However, the result of the PM muscle was partially different from that of previous studies on lateral differences in the CSA of the PM muscle among athletes [22, 23]. In a previous study on young male professional tennis players, the volume of the iliopsoas muscle on the nondominant side was 13 % greater than that of the iliopsoas muscle on the dominant side. In non-athlete subjects, the iliopsoas muscle on the dominant side had only a 4 % greater volume than the iliopsoas muscle on the non-dominant side [23]. Track and field athletes demonstrated lateral differences in thigh muscle sizes but no differences in PM muscle sizes. However, the causal relationships between them are yet to be confirmed.

The larger outer PM muscle could affect the swing of the outer leg. A previous study showed that a faster forward return in the swing phase of the outer leg caused a positive correlation between hip flexion strength and sprint performance [24]. From a model-based calculation, the iliopsoas muscle was the major contributor to hip flexion torque at running speeds above 7.0 ms^−1^ [20]. It is reasonable to assume that the PM muscle is important for fast sprint running, as it is the largest hip flexor muscle [25]. In straight running, however, faster top speeds are achieved by applying greater support forces during the contact phase rather than by repositioning the limbs rapidly during the swing phase [26]. Curve running is different from straight running because the speed in curve running is generally slower than the speed in straight running, and asymmetric lower extremity kinematics exist in curve running [2, 6]. Future work on joint kinetic and/or kinematic data should be undertaken to explain the effect of the PM muscle on curve running.

Significant correlation was observed between averaged sprint time along a curve and total muscle volume of PM, QF, and HM (Table 2). This is similar result with previous study which 100-m sprint time was correlated with CSA of PM [14]. Then, lager CSA of PM is a factor in running shorter time in sprint race. However, symmetry index of sum of the PM, QF, and HM were not significantly correlated with averaged sprint time along a curve, so that sprint performance level was not significantly correlated with lateral difference of PM, QF, and HM.

Interpretation of the findings of this study could have been limited by the small sample size. This study compared the counterclockwise and clockwise sprinting times, even though the track and field athletes in this study did not compete at high levels. However, the participants including middle-distance runners and 400-m sprinters usually run around the track so that they could run at maximal effort although they rated their performance worse in opposite direction curve running. Otherwise, the lateral differences of the PM muscle could have affected the cross-directional difference in sprint time for these participants. It must be pointed out that all participants were right-leg dominant, which could have influenced the observed lateral differences in the CSA of the QF and HM muscles. Further studies could use a control group of left-leg dominant athletes to verify whether the inter-limb differences observed between the counterclockwise direction and clockwise direction were significant. Nevertheless, we believe that the data presented are valuable and that future research should address these limitations.

## Conclusions

The present results suggest that the cross-directional difference in sprint time is related to the lateral difference in the PM muscle. In curve sprinting, the PM muscle of the outer leg plays a greater role than the PM muscle of the inner leg.
